# Calcium Trimetaphosphate-Loaded Electrospun Poly(Ester Urea) Nanofibers for Periodontal Tissue Engineering

**DOI:** 10.3390/jfb14070350

**Published:** 2023-06-30

**Authors:** Priscila T. A. Toledo, Caroline Anselmi, Renan Dal-Fabbro, Abdel H. Mahmoud, Alexandra K. Abel, Matthew L. Becker, Alberto C. B. Delbem, Marco C. Bottino

**Affiliations:** 1 Department of Cariology, Restorative Sciences, and Endodontics, School of Dentistry, University of Michigan, Ann Arbor, MI 48109, USA; priscilt@umich.edu (P.T.A.T.); caanselm@umich.edu (C.A.); renandf@umich.edu (R.D.-F.); abdelm@umich.edu (A.H.M.); 2 Department of Preventive and Restorative Dentistry, School of Dentistry, São Paulo State University (UNESP), Araçatuba 16015-050, SP, Brazil; alberto.delbem@unesp.br; 3 Department of Morphology and Pediatric Dentistry, School of Dentistry, São Paulo State University (UNESP), Araraquara 14801-385, SP, Brazil; 4 Departments of Chemistry, Mechanical Engineering and Material Science, Orthopaedic Surgery, Duke University, Durham, NC 27708, USA; aabel@email.unc.edu (A.K.A.); matthew.l.becker@duke.edu (M.L.B.); 5 Department of Biomedical Engineering, College of Engineering, University of Michigan, Ann Arbor, MI 48109, USA

**Keywords:** nanofibers, tissue engineering, calcium, phosphates, poly(ester urea)

## Abstract

The objective of this research was to create and appraise biodegradable polymer-based nanofibers containing distinct concentrations of calcium trimetaphosphate (Ca-TMP) for periodontal tissue engineering. Poly(ester urea) (PEU) (5% *w*/*v*) solutions containing Ca-TMP (15%, 30%, 45% *w*/*w*) were electrospun into fibrous scaffolds. The fibers were evaluated using SEM, EDS, TGA, FTIR, XRD, and mechanical tests. Degradation rate, swelling ratio, and calcium release were also evaluated. Cell/Ca-TMP and cell/scaffold interaction were assessed using stem cells from human exfoliated deciduous teeth (SHEDs) for cell viability, adhesion, and alkaline phosphatase (ALP) activity. Analysis of variance (ANOVA) and post-hoc tests were used (α = 0.05). The PEU and PEU/Ca-TMP-based membranes presented fiber diameters at 469 nm and 414–672 nm, respectively. Chemical characterization attested to the Ca-TMP incorporation into the fibers. Adding Ca-TMP led to higher degradation stability and lower dimensional variation than the pure PEU fibers; however, similar mechanical characteristics were observed. Minimal calcium was released after 21 days of incubation in a lipase-enriched solution. Ca-TMP extracts enhanced cell viability and ALP activity, although no differences were found between the scaffold groups. Overall, Ca-TMP was effectively incorporated into the PEU fibers without compromising the morphological properties but did not promote significant cell function.

## 1. Introduction

Periodontal disease, a multifactorial inflammatory disorder affecting the supporting tissues of the teeth, poses a significant health burden worldwide. Over half of the global population is estimated to suffer from some form of periodontal disease, making it one of the most prevalent oral health conditions. This chronic condition leads to tooth loss and has profound implications for overall systemic health [1,2]. Conventional treatment modalities such as scaling, root planing, and surgical interventions have shown limited success in achieving complete regeneration of periodontal tissues. In recent years, tissue engineering has emerged as a promising approach to address the limitations of conventional therapies by leveraging the principles of stem cell biology, biomaterial science, and molecular biology [3].

Numerous techniques have emerged to regenerate compromised periodontal tissues, especially using membranes as scaffolds [4]. For clinical applications, an optimal membrane should fulfill specific criteria. These criteria encompass biocompatibility and biodegradability, as well as providing a suitable surface and a porous three-dimensional framework that facilitates cell migration, attachment, infiltration, proliferation, and differentiation. Additionally, the membrane should possess sufficient mechanical strength to support tissue regeneration by maintaining the space [4]. This way, various biomaterials can be chosen to manufacture barrier membranes, including synthetic and natural materials. Along with selecting the right biomaterial, it is crucial to use appropriate manufacturing methods when developing a membrane [5]. Electrospinning is a versatile and user-friendly technique. It is widely used to produce nanofibrous scaffolds with a high surface area-to-volume ratio, facilitating cell attachment and proliferation [6]. They can also be made with or without incorporating therapeutic agents for treating infections, controlling inflammation, modulating cell differentiation, and promoting tissue regeneration [6,7].

PEU, which stands for poly(ester urea), is a promising polymer in the biomedical field due to its biodegradability, biocompatibility, and tunability. These materials have found extensive application in tissue engineering and various medical fields. Notably, their versatility makes them particularly suitable for repairing periodontal defects, as they exhibit excellent compatibility with soft and hard tissues [8,9,10,11]. PEU is a copolymer comprising three building blocks: an aliphatic diol or diamine, a diisocyanate, and a dicarboxylic acid or its anhydride. Combining these building blocks forms ester and urea linkages in the polymer backbone. By varying its composition, the mechanical properties of PEU can be tuned broadly. For example, using longer or shorter chain diols or diamines can change the flexibility and stiffness of the polymer [12]. Similarly, using different amino acids can also influence the mechanical properties of the polymer [13]. Additionally, by adjusting the length of the diol or the amino acid’s hydrophilicity, the degradation rate can be easily controlled [12].

The addition of molecules as antibiotics and cations (Sr^2+^, Mg^2+^, Si^4+^, and/or Zn^2+^) to improve scaffolds’ biological or antimicrobial properties has been widely tested [14,15]. Due to their remarkable resemblance to the mineral components of bone and their exceptional osteoconductive and osteoinductive properties, calcium phosphates (CaP) have been widely employed in bone tissue engineering applications [16,17,18,19]. The crucial role of low calcium (Ca^2+^) ion concentrations in supporting cell proliferation, migration, and differentiation has been reported [17,18,19,20,21]. Meanwhile, sodium trimetaphosphate (STMP, Na_3_P_3_O_9_) is a cyclic polyphosphate working as an effective crosslinker, not showing toxicity at any concentration, and has been used in the food industry since being FDA-approved for humans [20]. Studies investigating the potential of STMP found that it provides nucleation sites for calcium phosphate apatite precipitation [21] since it leads to a more anionic surface [22,23]. When added to a polyamide-based scaffold, STMP produced higher elastic modulus, elongation at break, and tensile strength, in addition to generating a hydrophilic surface that facilitates cell adhesion, which is essential for bone repair [24].

Based on the ability of STMP to improve the biological and mechanical properties of polymers and the effects of calcium on bone mineralization, the replacement of sodium ions by calcium ions may generate a compound that combines the properties of both (TMP and calcium) on mineralization processes. Thus, to determine the effect of sodium replacement for calcium in the STMP on cell adhesion, proliferation, and differentiation, the present study incorporates different concentrations of Ca-TMP into PEU nanofibrous scaffolds and investigates the mechanical and biological properties of those scaffolds for periodontal tissue engineering.

## 2. Materials and Methods

### 2.1. Materials

We obtained 1,1,1,3,3,3-hexafluoro-2-propanol (HFP) from Sigma-Aldrich in St. Louis, MO, USA. The calcium trimetaphosphate (Ca-TMP) used had a molecular weight of 594.07 and a particle size of 236 ± 50 nm. It was synthesized in the Laboratory of Pediatric Dentistry of the Department of Preventive and Restorative Dentistry at São Paulo State University (FOA/UNESP). The alpha-minimum essential medium (α-MEM), fetal bovine serum (FBS), and penicillin/streptomycin were procured from Gibco in Grand Island, NY, USA. Invitrogen in Waltham, MA, USA, provided AlamarBlue^®^ (DAL1100), ActinRed™ 555, and ActinGreen™ 488. Additionally, we used the SensoLyte p-nitrophenyl phosphate (pNPP) ALP kit from AnaSpec, Inc. in Fremont, CA, USA.

### 2.2. PEU (Poly Ester Urea) Synthesis

PEU (poly ester urea) 50% PHE6 P(1-VAL-10) or 50% P6V10: M(1-PHE-6) (50.0 g, 0.07 mol, 0.5 eq.), M(1-VAL-10) (47.4 g, 0.07 mol, 0.5 eq.), sodium carbonate (43.4 g, 0.41 mol, 3.1 eq), triphosgene (15.7 g, 0.05 mol, 0.4 eq.) (M_n_ = 69.9 kDa, M_w_ = 134.4 kDa, Đ_M_ = 1.9, T_g_ = 38.4 °C, T_d_ = 235.2 °C) was produced via an interfacial step growth polymerization as described previously [9,25]. In summary, an interfacial polymerization process was carried out by combining chloroform and water. In a 2 L reactor equipped with an overhead mechanical stirrer, the desired molar equivalents (1 eq., in total) of di-p-toluenesulfonic acid monomer salts and sodium carbonate (3.15 eq.) were introduced. Around 500 mL of hot distilled water (100 °C) was added to the flask, and the solution was stirred at a rate of 200 RPM until complete dissolution. Subsequently, the flask was cooled in an ice bath to achieve a temperature of 10 °C. Once cooled, triphosgene (0.43 eq.) was dissolved in 500 mL of chloroform and added to the solution. Immediate whitening of the solution occurred upon adding the organic solution, which was stirred for 48 h at 400 RPM. The resulting solution was transferred to a separatory funnel and washed with 500 mL of deionized water. The chloroform layer was precipitated dropwise into 4 L of hot water. In the case where residual salt peaks were detected in the H-NMR analysis, the crude product was dissolved in a volume of acetone measuring 2 L and subsequently precipitated into hot water with a volume of 4 L. This process was carried out to effectively eliminate any remaining salts present. ^1^H NMR (500 MHz, 303 K, DMSO-d6, from X = 6 from valine monomer component, Y = 2 from phenylalanine monomer component) δ = 0.81–0.85 (m, 12H, -CH(CH_3_)_2_), 1.16–1.19 (b, 2YH, -COOCH_2_CH_2_(CH_2_)_Y_CH_2_CH_2_OOC-), 1.18–1.26 (br, 2XH, -COOCH_2_CH_2_(CH_2_)_X_CH_2_CH_2_OOC-) 1.43–1.44 (b, 4H, -COOCH_2_CH_2_(CH_2_)_Y_CH_2_CH_2_OOC-), 1.53–1.54 (br, 4H, -COOCH_2_CH_2_(CH_2_)_X_CH_2_CH_2_OOC-), 1.95–1.98 (m, 2H, -CH(CH_3_)_2_), 2.50 (s, DMSO), 2.90–2.92 (m, 2H, -NHCH(CH_2_Ph)COO-), 3.29–3.33(H_2_O), 3.95 (t, 2H, -COOCH_2_CH_2_(CH_2_)_Y_CH_2_CH_2_OOC-),4.03–4.06 (m, 6H, -CHCOOCH_2_(CH_2_)_X_CH_2_OOCCH-) 4.36–4.39 (m, 2H, -NHCH(CH_2_Ph)COO-), 6.36–6.38 (d, 2H, -NH-), 6.48–6.52 (d, 2H, -NH-), 7.13–7.25 (m, 10H, -C6H_5_) ppm (Figure S1).

### 2.3. Cell/Ca-TMP Interaction

Previously isolated and characterized SHEDs were generously donated by Dr. Jacques Nör (University of Michigan, School of Dentistry, Ann Arbor, MI, USA) and were used to assess cell function in response to Ca-TMP powder. SHEDs were cultured in an α-MEM. The medium was supplemented with 15% FBS and 1% penicillin/streptomycin. SHEDs at passages 4–6 were maintained in a humidified atmosphere at 37 °C with 5% CO_2_ and utilized for the experimental procedures. To determine a non-cytotoxic concentration to be incorporated into the PEU fibers, solutions containing different concentrations of Ca-TMP (10, 25, 50, 100, 150, and 200 µM) were placed in contact with SHEDs for 1, 3, and 5 days. The cells were seeded in a density of 1 × 10^4^ cells/well in 96-well plates and incubated at 37 °C and 5% CO_2_ for 24 h to the initial adhesion. After that, the Ca-TMP-containing media was added, and this media was refreshed every two days. Cell viability was assessed at each time point using 10% alamarBlue^®^. After 3 h of incubation, the fluorescence was measured at 560 nm excitation and 590 nm emission (Spectra Max Id3). The fluorescence values were transformed in percentages using the average of the negative control group (α-MEM without FBS) as 100%. For the qualitative analysis of cell adhesion and spread, cells were fixed using 4% paraformaldehyde, and the actin filaments were stained with ActinRed™ 555.

### 2.4. Fabrication of PEU and PEU Matrices Loaded with Ca-TMP

To prepare four different polymer solutions, PEU was dissolved in hexafluoropropylene (HFP) at a concentration of 5% (*w*/*v*). These solutions were stirred overnight. Subsequently, the PEU solutions were loaded with different concentrations (0, 15, 30, 45 wt%, relative to the total polymer weight) of Ca-TMP particles. To ensure that the particles were evenly spread within the polymer solution, the mixtures were stirred for 24 h and sonicated for 30 min. Next, the solutions were individually loaded into plastic syringes (Becton, Dickson and Company, Franklin Lakes, NJ, USA) coupled with 27 G metallic blunt-tip needles (CML Supply, Lexington, KY, USA). The process of electrospinning was carried out at room temperature with specific parameters. These included a fixed spinning distance of 20 cm, a rotating collector (120 RPM) with a diameter of 2.5 cm and a peripheral speed of 0.15 m/s, a flow rate of 1.5 mL/h, and electric voltages of 16 kV. The approximate time of the electrospinning session was 2.6 h for each group. The electrospinning system consisted of a high-voltage source (ES50P-10W/DAM, Gamma High Voltage Research Inc., Ormond Beach, FL, USA), a syringe pump (Legato 200, KD Scientific Inc., Holliston, MA, USA), and a grounded stainless steel collecting drum connected to a high-speed mechanical stirrer (BDC6015, Caframo Limited, Georgian Bluffs, ON, Canada). After the electrospinning process, the mats produced, known as matrices, underwent a minimum of 48 h of vacuum drying to eliminate any remaining solvent [26,27].

### 2.5. Characterization of the Morphology and Composition of the Electrospun Fibers

The morphology and general structure of the electrospun fibers were assessed using a field-emission scanning electron microscope (TESCAN MIRA3, Kohoutovice, Czech Republic). Samples (*n* = 3) from each group were affixed onto an aluminum stub, coated with a thin layer of gold–palladium through sputter-coating, and imaged at an acceleration voltage of 5 kV. Fiber diameter distribution was subsequently determined using Image J software (version 1.53, National Institutes of Health, Bethesda, MD, USA). Measurements were conducted on 50 individual fibers per image acquired (three images per group) at a consistent magnification of 5000×. The percentage of porosity of the scaffolds was calculated from three images of each scaffold at a 5000× magnification using the same software. In addition, energy dispersive X-ray spectroscopy (EDS) analysis was carried out under SEM (*n* = 3) to obtain semi-quantitative data regarding the chemical composition of the fibers.

The chemical properties and Ca-TMP incorporation in all matrices were assessed (*n* = 2) using Fourier-transform infrared spectroscopy (FTIR) in attenuated total reflection mode (Nicolet iS50, Thermo Fisher Scientific, Waltham, MA, USA). The FTIR measurements were performed over a range of 700–4000 cm^−1^ at a resolution of 4 cm^−1^, with 64 scans conducted in random regions. X-ray diffraction (XRD) was used to analyze the crystalline phase composition (*n* = 2) using a Rigaku Ultima IV diffractometer (Rigaku Americas Corporation, Woodlands, TX, USA) in the Bragg–Brentano geometry. Cu Kα radiation with a wavelength (λ) of 1.54 Å was utilized. Scans were conducted in the 2θ range from 5° to 45°, with a step size of 0.05° and a scanning speed of 1°/min. Rigaku’s data analysis software (PDXL Version 2.6.1.2) and the Inorganic Crystal Structure Database (ICSD) were employed for phase identification. Thermogravimetric analysis (TGA) of the fibers was performed (*n* = 2) using a simultaneous thermal analyzer (SDT Q600, TA Instruments, Dallas, TA, USA). The heating rate was set at 10 °C/min. Three fibers from each group were subjected to a step-by-step program under a pure nitrogen (N2) environment. This program involved holding the temperature at 50 °C for 1 min, heating it from 50 to 650 °C at a rate of 10 °C/min, and finally holding it at 650 °C for 2 min. Data were recorded after the final step.

### 2.6. Contact Angle

Fibers produced by electrospinning from pure PEU and Ca-TMP-loaded solutions were then affixed onto microscope glass coverslips (*n* = 4) sourced from Fisherbrand (Fisher Scientific UK Ltd., Loughborough, UK). A goniometer (M200 Rame-Hart Goniometer, Succasunna, NJ, USA) was employed to determine the surface contact angle of the fibrous matrices. Three consecutive drops of distilled water, each approximately 1 μL in volume, were placed on each sample. The contact angles of the drops were measured and subsequently averaged to obtain a representative value.

### 2.7. Mechanical Properties, Swelling, and Degradation Profile

The mechanical performance (e.g., elongation at break, tensile strength, Young’s modulus, yield stress, and yield strain) of all the matrices was evaluated using uniaxial tensile testing on an eXpert 5601 machine from ADMET (Norwood, MA, USA) using a load cell from the same company (SM-250-961—250 lbf) of 1 kN. Rectangular samples with dimensions of 25 × 3 mm^2^ were utilized for the tests, with a gauge length of 19 mm and eight samples per group examined. The testing was conducted in two ways: dry, which means the samples were tested immediately without any storage, and wet, which means the samples were stored in a PBS solution for 24 h before being tested. The crosshead speed during testing was set at 1 mm/min. To determine the thickness of each sample, a digital caliper (Mitutoyo Corp., Kanagawa, Japan) was used to take measurements at three separate locations. We collected mechanical data from the stress-strain curves of each sample and presented the results as the average value along with the standard deviation (SD).

### 2.8. Swelling and Degradation Profile

To determine the water retention capability of the membranes, the fibers (1 cm × 1 cm; *n* = 4/group) were incubated in PBS at 37 °C for 24 h. The wet and dry weights of the specimens were determined before and after incubation at 37 °C. The changes in the fiber’s weight between the wet (Ww) and dry (Wd) states were used to determine the volumetric swelling through the following equation:

Swelling ratio=(Ww−Wd)/Wd × 100

The in vitro degradation of the manufactured fibers was assessed by immersing 10 × 10 mm electrospun fibers (*n* = 4) in solutions containing PBS and lipase (150 U/L) at 37 °C while continuously monitoring changes in their weight over a period of time (91 days) [28]. At predetermined time intervals, the samples were taken out of the incubation medium, lightly dried using low-lint wipes manufactured by Kimberly Clark Corporation (Kimwipes, Irving, TX, USA), rinsed twice with deionized water, and vacuum-dried for 24 h before recording their weights. The degradation profile was determined using the following formula: Wt represents the remaining weight at a given time, and W0 denotes the initial dried weight.

Degradation ratio (%)=Wt/W0 × 100

### 2.9. Calcium Release Assay

Calcium release from particles was studied in duplicates by immersing 1 cm^2^ of the membranes from PEU + 45% Ca-TMP and pure PEU groups (*n* = 3) in 1 mL of PBS and lipase at 37 °C. During the 21-day period, we used the supernatant to measure calcium levels using the calcium colorimetric assay kit (MAK022, Sigma Aldrich) according to the manufacturer’s instructions. This kit detects the chromogenic complex formed between calcium ions and o-Cresolphthalein to determine the concentration of calcium ions. The absorbance, which is directly proportional to the concentration of calcium ions present in the sample of this complex, was read at 575 nm on a plate reader (Spectra Max Id3, Molecular Devices LLC, San Jose, CA, USA), and it was calculated from a standard calcium solution prepared in parallel. The values obtained by reading the PEU group were used as background and subtracted from the PEU + 45% Ca-TMP group to obtain the actual reading.

### 2.10. Cell/Scaffold Interaction

SHEDs were also used to assess cell function in response to the formulated PEU/Ca-TMP fibers using the same cell culture conditions described in 2.2. To evaluate the cell response in contact with PEU/Ca-TMP fibers, all electrospun matrices were cut into 15 × 15 mm^2^ samples and disinfected in a laminar flow hood by UV light (1 h on each side), then adapted into 24-well plates with metal rings. SHEDs were seeded at a density of 30,000 cells/matrix and incubated for 1 h to allow initial cell adhesion on the samples. As mentioned, cell viability was assessed using alamarBlue^®^ after 1, 3, and 7 days. Fibers of PEU without Ca-TMP on day 1 were used as the control [29,30]. As previously mentioned, the adhesion and spreading of SHEDs on scaffold surfaces were assessed at 1, 3, and 7 days with ActinGreen™ 488. Matrix surfaces (*n*  =  4) were analyzed under direct fluorescence microscopy (Echo Revolve, BICO Company, San Diego, CA, USA) at a 10× magnification.

### 2.11. Alkaline Phosphatase (ALP) Activity

To induce osteoblastic differentiation in SHEDs treated with 50 µM of Ca-TMP (Section 2.2) and cells seeded on the fibers (Section 2.9), two types of media were employed: osteogenic differentiation medium (OM) and basal medium. OM consisted of alpha-MEM media supplemented with 100 nM dexamethasone, 10 mM β-glycerophosphate, 50 μg/mL ascorbate phosphate, and 10% FBS. ALP (alkaline phosphatase) activity measurements were obtained by collecting cell lysates at 7 and 14 days for cells seeded on membranes and at 14 days only for cells/Ca-TMP interaction. The SensoLyte pNPP ALP kit was utilized according to the manufacturer’s instructions for ALP activity detection. Briefly, the cells were suspended in 500 μL of Triton X-100, and the resulting cell homogenates were centrifuged at 2500× *g* for 10 min at 4 °C. The supernatants obtained were used in the ALP assay, where pNPP served as the phosphatase substrate, and the kit-provided ALP acted as the standard. Controls consisted of media or PEU fibers lacking Ca-TMP. Absorbance at 405 nm was measured using a microplate reader (SpectraMax iD3) to quantify the ALP activity.

### 2.12. Statistical Analysis

Data were submitted to normality (Shapiro–Wilk) and homogeneity of variance (Levene) tests. Cell viability, fiber diameter, contact angle, and mechanical test data were analyzed using one-way ANOVA followed by Tukey or Games–Howell post-hoc. Cell viability on scaffolds and ALP activity data were analyzed using two-way ANOVA followed by Sidak post-hoc test. Fluorescence images were analyzed qualitatively. All the experiments were performed, on at least two different occasions, to verify their reproducibility. Statistical inferences were based on a 5% significance level.

## 3. Results

### 3.1. Cell/Ca-TMP Interaction

**Figure 1:**
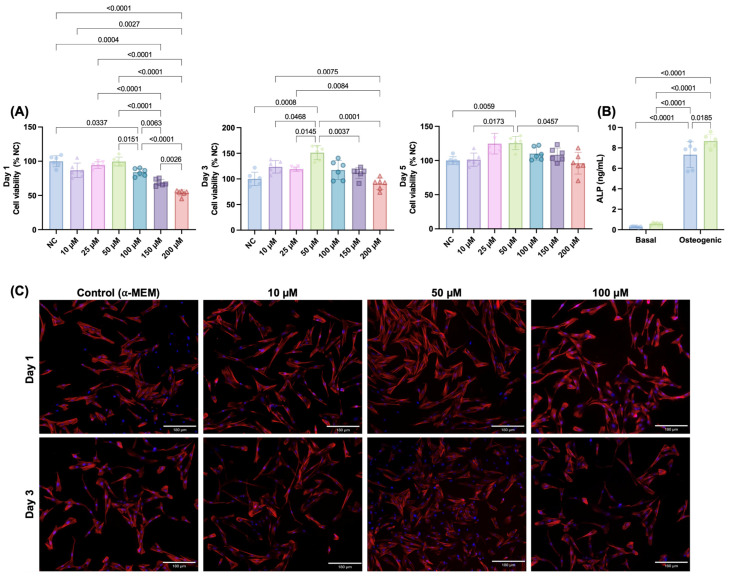
Initial screening for Ca-TMP (10, 25, 50, 100, 150, and 200 µM) in contact with SHEDs. (**A**) Percentage of cell viability (AlamarBlue^®^) normalized by the negative control (NC) within each time point. Welch’s ANOVA/Games–Howell; α = 5%. (**B**) Alkaline phosphatase (ALP) activity of SHEDs cells after 14 days. Data presented as mean ± SD. Two-way ANOVA/Tukey; α = 5%. (**C**) Representative fluorescence images of SHEDs cells at days 1 and 3 stained by F-actin (ActinRed™ 555).

To determine the cytocompatibility of pure Ca-TMP, we first evaluated the viability of several increasing concentrations on SHEDs cells before including it in membrane manufacturing. Initially, all concentrations decreased cell viability in a dose-dependent way for 50 µM to 200 µM amounts. However, cell viability increased from day 1 to day 3 for all tested concentrations, with 50 µM showing the significantly highest viability. From day 3 to day 5, there was a minor decrease in cell viability for all groups, but the 50 µM kept showing as the best concentration (Figure 1A). Based on this, we used this amount of Ca-TMP to evaluate the alkaline phosphatase activity. The results of ALP activity are shown in Figure 1B. After 14 days of culture, the Ca-TMP applied to the osteogenic media promoted a significantly higher activity when compared to osteogenic media only. The qualitative cell adhesion and spread staining results are consistent with the quantitative findings, indicating no hindrance in cell spreading caused by the concentration of Ca-TMP (Figure 1C). To determine the particle size, a SEM analysis was performed. As depicted in Figure S1B, the particles ranged from 171 nm to 320 nm with an average size of 236 ± 50 nm.

### 3.2. Morphological and Chemical Characterizations of the Fibers

**Figure 2:**
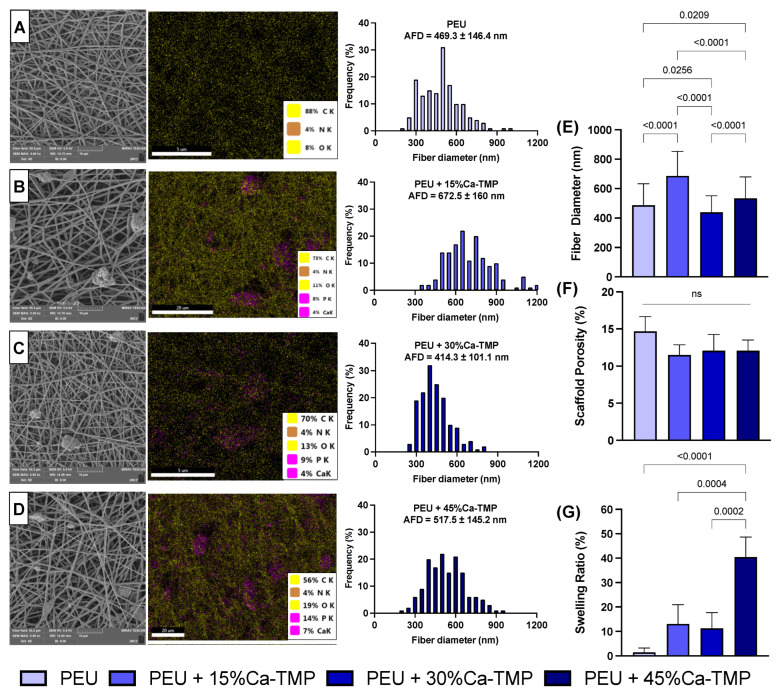
Evaluation of chemical–morphological membranes. (**A**–**D**) Scanning electron microscope (SEM) images, energy dispersive X-ray spectroscopy (EDS—5000× magnification), and histogram of fiber diameter of PEU (control), 5% PEU + 15% Ca-TMP, 5% PEU + 30% Ca-TMP, and 5% PEU + 45% Ca-TMP, respectively. (**E**) Fiber diameter, (**F**) scaffold porosity, and (**G**) swelling ratio evaluations (mean ± SD, *p* < 0.05).

**Figure 3:**
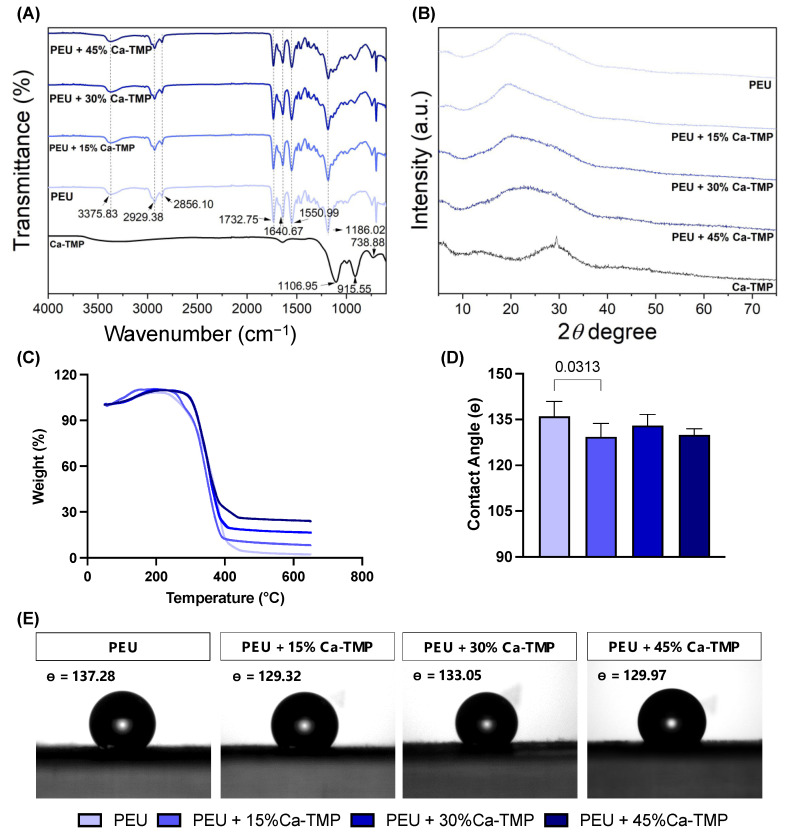
Evaluation of chemical–morphological membranes. (**A**) Fourier-transform infrared spectroscopy (FTIR) spectra, (**B**) X-ray diffraction (XRD), (**C**) thermogravimetric analysis (TGA) of electrospun fibers, and (**D**) contact angle data presented as mean ± SD, and (**E**) images formed between water and the PEU- or PEU/Ca-TMP-based membranes containing different concentrations.

The addition of Ca-TMP produced thicker fibers than pure PEU, except for the PEU + 30% Ca-TMP group, which presented a thinner fiber diameter. Also, while the pure fibers were visually smooth (Figure 2A), the SEM revealed Ca-TMP nanoparticle precipitates present in the fibers, differing in shape and amount, within a nano-sized scale, which was confirmed by the EDS analysis (Figure 2B–D). The PEU and PEU/Ca-TMP-based membranes presented fiber diameters at 469 nm and 414–672 nm, respectively (Figure 2E). There were no significant differences (*p* > 0.05) in the percentage of porosity regardless of the presence and concentration of Ca-TMP (Figure 2F). Lastly, the FTIR analysis also revealed Ca-TMP incorporation into the membranes and PEU into the PEU-based membranes (Figure 3A). The broad stretch at 3350–3500 cm^−1^ is for the N-H (urea), and the stretch at 1735 cm^−1^ is characteristic of the C=O (ester) bond stretching and confirms the presence of an ester bond. The stretch at 1650–1690 cm^−1^ is typical of the C=O (urea) bond stretching from the urea and confirms the presence of the urea bond. The stretch around 1000–1300 cm^−1^ represents the C-O (ester) bond and the C-H (alkyl) bending. All these characteristic signals confirm that we had successfully synthesized the poly(PHE6XX-VAL-10XX) PEU [31].

The X-ray diffraction pattern (Figure 3B) of Ca-TMP can be described as a reflection at 29.45 degrees with FWHM of 0.35 degrees sitting on an amorphous halo, which is the major portion of Ca-TMP that is amorphous with an estimated content of 93 wt%. The d-spacing of the crystalline phase is 3.03 angstrom based on Bragg’s law, and the grain size is estimated to be around 23.5 nm from the peak width according to the Scherrer equation. The as-synthesized PEU shows an amorphous halo in the range from 15 to 37 degrees, which overlaps several diffractions, with the major ones at 19.6 and 23.9 degrees. The PEU-Ca-TMP profiles can be roughly viewed as the linear combination of PEU and Ca-TMP. The diffraction around 29.45 degrees is significantly weakened in these composites, suggesting the interaction between PEU and Ca-TMP disrupted the crystallization of Ca-TMP.

The thermal gravimetric analysis (TGA) of the nanometer fibers also evidenced the efficient incorporation of Ca-TMP, showing the thermal stability of the polymer up to 250 °C. Above that temperature, a similar weight-loss pattern was observed for all samples (Figure 3C). The degradation temperatures were detected around 250 °C, 260 °C, 285 °C, and 285 °C for PEU, PEU + 15% Ca-TMP, PEU + 30% Ca-TMP, and PEU + 45% Ca-TMP fibers, respectively. When PEU and Ca-TMP interact, it can alter the molecule’s structure. This can lead to stronger connections between scaffold polymeric chains, resulting in improved thermal stability and higher degradation temperatures.

### 3.3. Contact Angle

Regardless of the Ca-TMP content, all PEU-based membranes had a hydrophobic surface with a contact angle greater than 90°, indicating low wettability (*p* < 0.05). The PEU/Ca-TMP membranes containing 15, 30, and 45% of Ca-TMP nanoparticles revealed a less hydrophobic surface than the pure PEU membrane, with just the 15% Ca-TMP one being statistically different (*p* < 0.05) (Figure 3D,E).

### 3.4. Mechanical Properties, Swelling, and Degradation Profile

**Figure 4:**
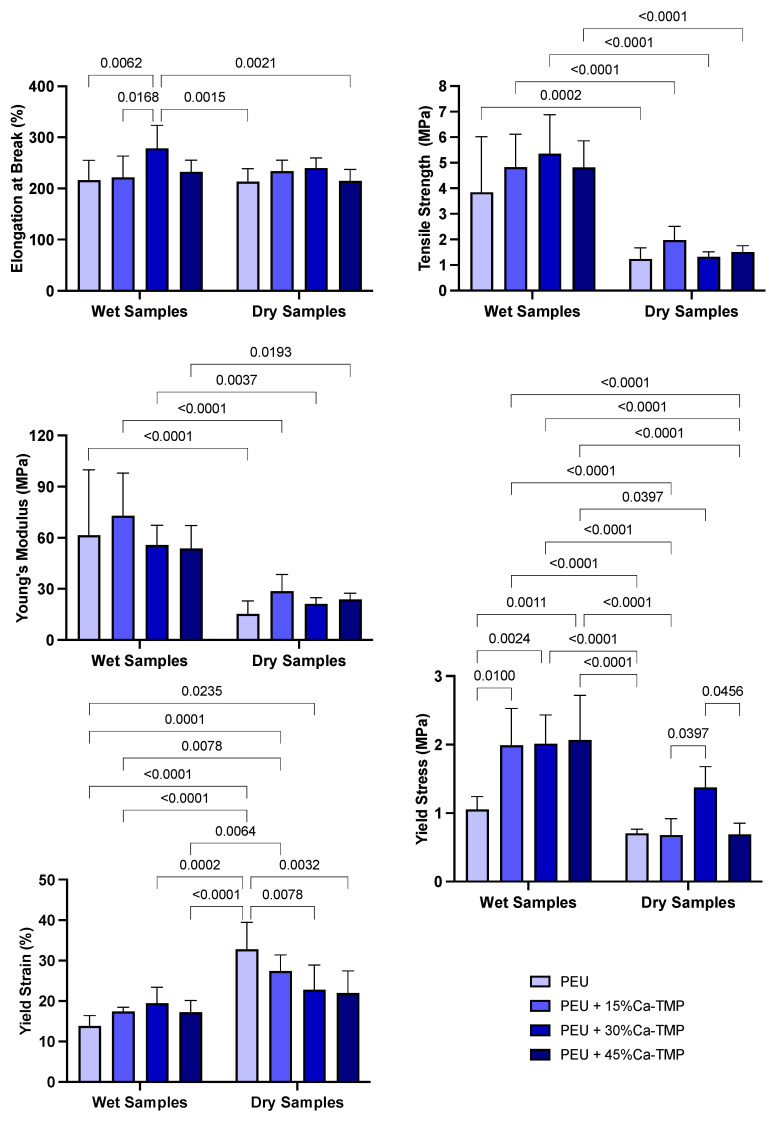
Mechanical characterization of membranes. Elongation at break (%), tensile strength (MPa), Young’s modulus (MPa), yield stress (MPa), and yield strain (%) graphs of dry and wet PEU fibers loaded with Ca-TMP. Data presented as (mean ± SD). Two-way ANOVA/Tukey; α = 5%. Significant interaction between variables for yield stress (MPa) and yield strain (%), *p*-value = 0.0037 and 0.0004, respectively.

The average specimen thickness for each group was as follows: PEU = 0.25 mm, PEU + 15%Ca-TMP = 0.27 mm, PEU + 30%Ca-TMP = 0.28 mm, and PEU + 45%Ca-TMP = 0.30 mm. The mechanical performance (e.g., elongation at break, tensile strength, Young’s modulus, yield stress, and yield strain) of the fabricated nanofiber membranes in wet and dry conditions are presented in Figure 4. The PEU membranes showed a soft nature with moderate flexibility regardless of the storage condition. There were no notable differences in elongation at break between the fibers made from PEU, PEU + 15%Ca-TMP, and PEU + 45%Ca-TMP, in both dry and wet conditions (*p* > 0.05). However, the PEU + 30%Ca-TMP group displayed an increase in elongation compared to the PEU and PEU + 15%Ca-TMP groups in wet conditions and compared to the PEU and PEU + 45%Ca-TMP groups in dry conditions (*p* < 0.05). The groups observed a significant difference (*p* < 0.05) in the tensile strength between wet and dry conditions, increasing tensile strength in the wet state. In terms of Young’s modulus, all the groups demonstrated an increase in modulus and a significant difference in the wet versus dry conditions (*p* < 0.05), except for the comparison between the PEU + 45%Ca-TMP group in the wet state and the PEU + 15%Ca-TMP group in the dry state (*p* > 0.05).

The strain–stress curve showed that the yield stress was 1.05 ± 0.16 MPa for mats made of pure PEU in the wet condition. However, mats loaded with 15/30/45% Ca-TMP and PEU had higher yield stress values of 1.99 ± 0.46, 2.01 ± 0.38, and 2.06 ± 0.59 MPa, respectively. This indicates that adding Ca-TMP significantly increased the yield stress compared to the dry condition and even to PEU alone (*p* < 0.05). In the dry state, the yield stress of pure PEU was found to be 0.70 ± 0.05. For PEU mixed with 15%, 30%, and 45% Ca-TMP, the yield stress was 0.67 ± 0.21, 1.37 ± 0.27, and 0.69 ± 0.14 MPa, respectively. There was no significant difference between the groups, except for the PEU mixed with 30% Ca-TMP. The yield strain graph for PEU and PEU-loaded with 15/30/45% Ca-TMP electrospun mats showed that in wet conditions, the percentages were 13.79 ± 2.33, 17.38 ± 0.95, 19.43 ± 3.59, and 17.20 ± 2.66%, respectively. In dry conditions, the percentages were 32.82 ± 6.04, 27.40 ± 3.64, 22.77 ± 5.57, and 21.97 ± 4.98% for the PEU and PEU-loaded with 15/30/45% Ca-TMP, respectively. As a result, the Ca-TMP groups had a lower yield strain in wet conditions compared to dry conditions, with a significant difference in the PEU +15%Ca-TMP (*p* < 0.05).

**Figure 5:**
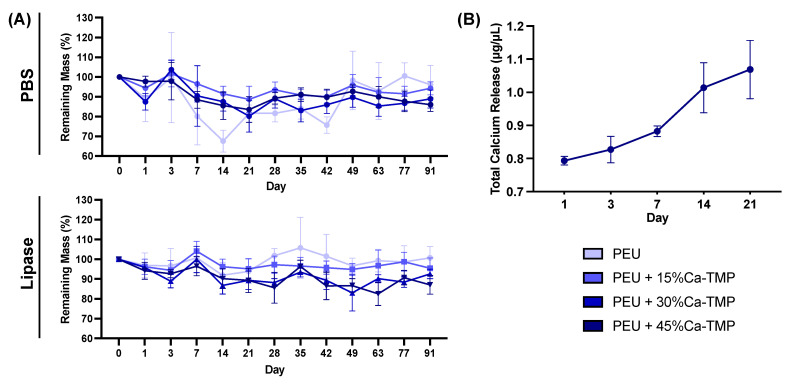
Membrane degradation and total calcium release quantification. (**A**) Degradation of electrospun membranes conditioned in PBS and lipase-enriched solutions over 91 days. Note the greater dimensional stability of the membranes even after 3 months of incubation. (**B**) Calcium ion release profile from the electrospun fibers immersed in a lipase-enriched solution derived over 21 days. Note the minimal calcium released from the fiber after 3 weeks of incubation. Data presented as (mean ± SD).

Based on the swelling profile results, it was observed that the introduction of Ca-TMP particles had an impact on the swelling properties of the fibers. The swelling percentage increased for PEU + 15%Ca-TMP and PEU + 30%Ca-TMP compared to pure PEU. Additionally, PEU + 45%Ca-TMP had the highest swelling ratio among all groups, which was statistically significant (Figure 2G). Regarding the degradation profile of the various PEU-based membranes, all groups demonstrated high stability through the degradation process (Figure 5A). Pure PEU presented ~96% and ~100% remaining mass by day 91, the PEU + 15% Ca-TMP fibers had about ~94% and ~95% of the remaining mass, followed by ~88% and ~92% for PEU + 30% Ca-TMP and ~85% and 87% for PEU + 45%Ca-TMP fibers, incubated in PBS and lipase, respectively. In this way, we demonstrated that adding Ca-TMP changed the degradation profile of the fibers in a concentration-dependent manner; however, it is important to mention that all groups maintained their stability from day 1 to day 91, with minimal variation in mass loss once they reached a plateau.

### 3.5. Calcium Release

The cumulative amount of calcium deposition was measured over 21 days and showed no calcium release for samples incubated in PBS. However, conditioning in lipase solution, the PEU + 45% Ca-TMP group showed a burst release of 0.793 ± 0.031 µg/µL after one day of incubation. For day 3, the values rose slightly to 0.821 ± 0.098 µg/µL, meaning that little new calcium was released, possibly due to the short time between readings (2 days). From day 7 to 21, the release of calcium increased in a higher fashion, reaching 0.882 ± 0.040 and 1.014 ± 0.184 µg/µL for days 7 and 14, respectively, peaking at day 21, with 1.069 ± 0.215 µg/µL (Figure 5B).

### 3.6. Cell/Scaffold Interaction

**Figure 6:**
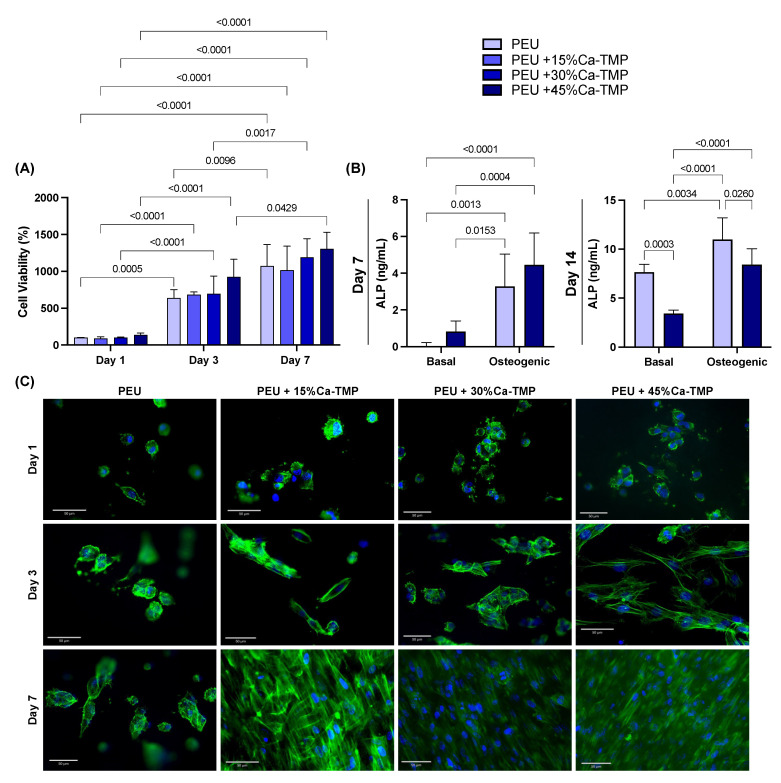
SHEDs cells and Ca-TMP electrospun membranes interaction. (**A**) Percentage of cell viability (AlamarBlue^®^) normalized by the PEU without Ca-TMP at day 1 value. Data presented as mean ± SD, *p* < 0.05. (**B**) Alkaline phosphatase (ALP) activity of SHEDs cells after 7 and 14 days. Repeated measures ANOVA/Sidak post-hoc; α = 5%. (**C**) Representative fluorescence images of SHED cells spread over the membranes at 1, 3, and 7 days stained by F-actin (ActinGreen™ 488).

Throughout the time points, all scaffolds’ formulations significantly increased intragroup cell viability (lowercase letters, *p* < 0.0001). However, when analyzing within the same time point, no differences were found regardless of the composition of the scaffold (uppercase letters, *p* > 0.05). Overall, all groups presented cell viability of ~100%, ~700%, and 1170% on days 1, 3, and 7, respectively (Figure 6A). All scaffolds containing Ca-TMP showed better cell adhesion when compared with pure PEU fibers, with intensified cell spreading observed for Ca-TMP-incorporated fibers at 7 days (Figure 6C). At day 7, the pure PEU and PEU + 45% Ca-TMP fibers cultivated in basal (−0.09 ± 0.06 and 1.02 ± 0.62 ng/mL) and osteogenic media (2.15 ± 0.05 and 4.46 ± 1.73 ng/mL) were different when comparing over media (lowercase letters, *p* < 0.05), but similar values within the same treatment (uppercase letters, *p* > 0.05). In a late analysis (day 14), pure PEU and PEU + 45% Ca-TMP fibers cultivated in basal (7.65 ± 0.80 and 3.44 ± 0.34 ng/mL) and osteogenic media (11.00 ± 2.20 and 8.41 ± 1.64 ng/mL) PEU + 45% Ca-MP resulted in a significant decrease compared to the pure PEU group regardless of the median conditioned (*p* < 0.0001) (Figure 6B).

## 4. Discussion

In this study, we extensively investigated the morphology and chemical composition of PEU membranes loaded with Ca-TMP, a calcium phosphate compound that has been explored for its potential use in biomedical applications due to its biocompatibility and ability to promote mineralization. In our study, we conducted an initial concentration screening of Ca-TMP to evaluate its cytocompatibility and determine the optimal concentrations to be incorporated into membranes. Our experiments found that the cells reacted positively to the material in a controlled environment. We noticed that specific concentrations of Ca-TMP were especially beneficial for the growth and multiplication of cells. Based on these results, we decided to include three concentrations of Ca-TMP in the PEU-based membranes.

According to the SEM micrographs, the membranes showed notable differences in fiber morphology, influenced by the polymer system and the incorporated Ca-TMP content. Membranes from the pure PEU group had more homogeneous fibers, while membranes based on PEU/Ca-TMP showed fibers with a larger diameter than PEU. Therefore, increasing Ca-TMP concentration resulted in an increased diameter, except for the group containing 30% Ca-TMP. This may be due to viscosity changes leading to stronger chain entanglement leading to a greater fiber formation [32]. Furthermore, the SEM images showed that the percentage of porosity did not change with incorporating Ca-TMP into the PEU scaffold formulation. Overall, different morphological characteristics may play a role in structural properties, such as degradation and mechanical properties. These two factors are critical aspects when considering the modification of the PEU materials [26].

Ca-TMP integration was confirmed through visual observation using SEM and identification using EDS. FTIR and XRD were subsequently used to verify the successful integration. Together, these results confirmed the chemical characteristics of the membranes synthesized in the study. When creating scaffolds, it is crucial to consider wettability. This can affect how cells attach, multiply, differentiate, and ultimately regenerate tissue once the scaffolds come into contact with moisture (i.e., body fluids present at the site of periodontal surgery). Many studies have shown that hydrophilic scaffolds have a higher affinity for cells, improving cell proliferation and wound healing [33,34]. According to our findings, all PEU-based membranes had a hydrophobic surface (i.e., contact angle greater than 90°), showing poor wettability [35]. However, when Ca-TMP was added to the PEU membrane, the contact angle was reduced, but not enough to transform the membrane from hydrophobic to hydrophilic, possibly due to the high hydrophobicity of sodium trimetaphosphate [22].

Despite the unfavorable hydrophobicity of the membranes, the scaffolds showed adequate morphology and chemical composition. For a membrane to be clinically useful, it needs to have satisfactory mechanical properties to prevent the collapse in periodontal defects as a result of compressive forces from soft tissues and mastication [6,36] and to resist cell adhesion and proliferation [6,36,37]. Considering these important characteristics, PEU was used due to its ability to be adjustable and controlled from the moment there is a change in the chemical composition of the compound. Our results showed that the mechanical properties of the PEU/Ca-TMP fibers were comparable to those of pure PEU in terms of elongation at break, except for the PEU +30%Ca-TMP. For the tensile strength and modulus of elasticity, all the groups exhibited high values in wet compared to dry conditions. Furthermore, in general, we observed significant differences in mechanical behavior between the dry and wet conditions for the scaffolds, even due to the high hydrophobicity of the material, which hindered the promotion of wettability on the surface of the membranes [6].

Although PEU is a material that offers great flexibility in modifying its amino acid chains to tune its mechanical properties, our work has shown that the PEU membranes we tested had a relatively low tensile strength, measuring around 1 MPa. To achieve optimal performance in periodontal regeneration, studies have suggested that a material with a tensile strength of 2 to 3 MPa, similar to the extracellular matrix (ECM), would be ideal [38]. The increase in stiffness and decrease in yield strain of electrospun membranes loaded with Ca-TMP in the wet condition compared to dry is probably because the fibers suffer from shrinkage after being kept at 37 °C for 24 h, promoting high stiffness and less elastic movement. Therefore, further modifications to the amino acid chains or other techniques may be necessary to enhance the mechanical properties of PEU fibers and optimize their performance for periodontal regeneration applications. Incorporating Ca-TMP into the material resulted in some improvement in its mechanical properties, but the observed differences were not significant enough to alter the overall mechanical behavior of the material.

A critical factor for scaffolds is their biodegradability. Typically, after implantation, the mechanical strength of membranes diminishes, and they lose their ability to serve as a physical barrier between tissues [27,39,40,41,42,43,44,45]. The degradation rate of a membrane is a critical factor in tissue regeneration, as these membranes need to remain functional for a minimum of 4 to 6 weeks to facilitate successful regeneration of the periodontal system and surface coverings. Moreover, the fibers’ degradation rate needs to match the tissue neoformation [3,40,46,47]. Consequently, having control over the degradation process of fibrous membranes becomes crucial in ensuring their efficacy. The present article shows that PEU and PEU/Ca-TMP fibers show high stability, with a low degradation rate of approximately > 80% for all groups after 3 months. This may have happened because, again, changing the PEU’s chemical composition alters its mechanical properties. However, it was not enough to promote greater degradability of the material [13].

Calcium is widely recognized as a crucial factor in osteoinduction, as it plays a significant role in affecting the growth and specialization of cells [48,49]. Successful incorporation of substances into the manufacture of membranes must also be accompanied by the ability to release them at an appropriate rate. Although we confirmed the successful incorporation of Ca-TMP into the fibers via several methods, our study revealed that the Ca-TMP-incorporated membranes exhibited insufficient degradability to effectively release calcium since, even for the PEU membrane loaded with the higher Ca-TMP tested (45%), minimal calcium was released after 21 days of incubation. This is related to the fact that the degradation rate was low, as previously described, showing high stability at day 91 and not being sufficient to promote high calcium release and stimulate the differentiation of cells [50]. Even though calcium (Ca^2+^) was not released in large amounts from the membranes, it is still important to evaluate its effectiveness, as it is an essential signal transduction element involved in regulating various cellular activities and is required at key stages of the cell cycle to ensure orderly progression and proliferation [51]. Whit this in mind, we investigated cell compatibility using human dental pulp stem cells (SHEDs), knowing their significant potential for osteogenic differentiation [52], key in periodontal bone regeneration. The results demonstrated no differences in cell viability for the four tested membranes over the three time points, mainly due to insufficient calcium released to stimulate cell proliferation [53,54]. The same happened with the alkaline phosphatase data; after seven days, the Ca-TMP group showed higher mineral differentiation activity but not enough to promote a significant difference between them and the osteogenic medium. However, although the scaffold did not promote osteogenic differentiation, it was clear that the membranes were not cytotoxic and even promoted better cell distribution and filopodia development.

In addition to PEU hindering degradation and drug release, the use of Ca-TMP seems to be another key factor in their unfavorable findings, as its synthesis is derived from STMP, which is used as a traditional scaffold crosslinker, responsible for the addition of intra- and intermolecular bonds at random locations in the polymer through an esterification reaction [55,56,57]. Although we worked with calcium trimetaphosphate, in the material synthesis process, sodium was removed, and calcium was added, maintaining its morphology [trimetaphosphate (P3O9)-3] [58]. Because of this, adding calcium to the trimetaphosphate could have promoted a crosslinking process in the PEU membrane, further increasing the material’s mechanical properties and not being suitable for promoting periodontal regeneration. However, the findings of this study must be considered despite the unexpected results since the polymer itself is subject to changes in its structure to improve its response, and the biomaterial has never had its cytotoxicity evaluated. Also, further investigations are essential to discover whether another polymer is favorable for Ca-TMP release and promotes periodontal regeneration.

## 5. Conclusions

The present study found that pure Ca-TMP powder at 50 µM concentration improved stem cells’ cell viability and ALP activity from human exfoliated deciduous teeth when they came in direct contact with it. Additionally, Ca-TMP-loaded PEU membranes were successfully made with suitable surface topography and mechanical properties. While the PEU membranes containing Ca-TMP did not negatively impact cell viability and proliferation, they did not affect osteogenic differentiation.

## Data Availability

The data presented in this study are available on request from the corresponding author.

## Supplementary Materials

The following supporting information can be downloaded at https://www.mdpi.com/article/10.3390/jfb14070350/s1, Figure S1: (A) 1H NMR spectra of 50% PHE6 P(1-VAL-10) with integration values and corresponding peak assignments. (B) Scanning electron microscope (SEM) image of the pure Ca-TMP powder and histogram showing the particle size distribution; Figure S2: force-strain and stress-strain curves for all groups in dry and wet conditions.
