# Supplementary material for: Calcium Trimetaphosphate-Loaded Electrospun Poly(Ester Urea) Nanofibers for Periodontal Tissue Engineering
Source: J Funct Biomater. 2023 Jun 30;14(7):350. doi: 10.3390/jfb14070350 (PMC10381820; doi:10.3390/jfb14070350)
Supplement: Supplementary file 1 [file jfb-14-00350-s001.zip › jfb-2442245-supplementary.pdf]

Supplementary Files

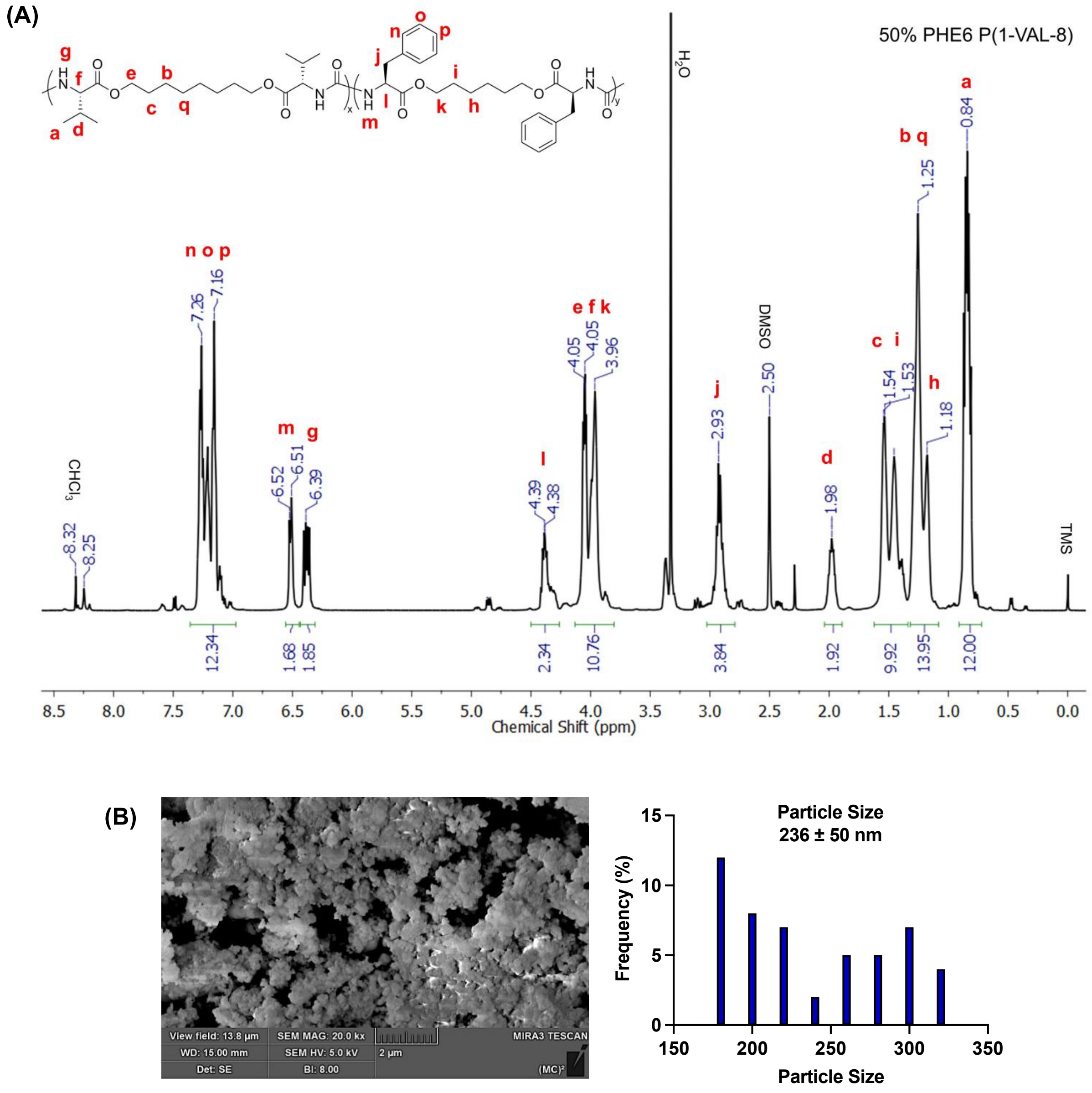

Figure S1: (A) ^{1}H NMR spectra of 50% PHE6 P(1-VAL-10) with integration values and corresponding peak assignments. (B) Scanning Electron Microscope (SEM) image of the pure Ca-TMP powder and histogram showing the particle size distribution.

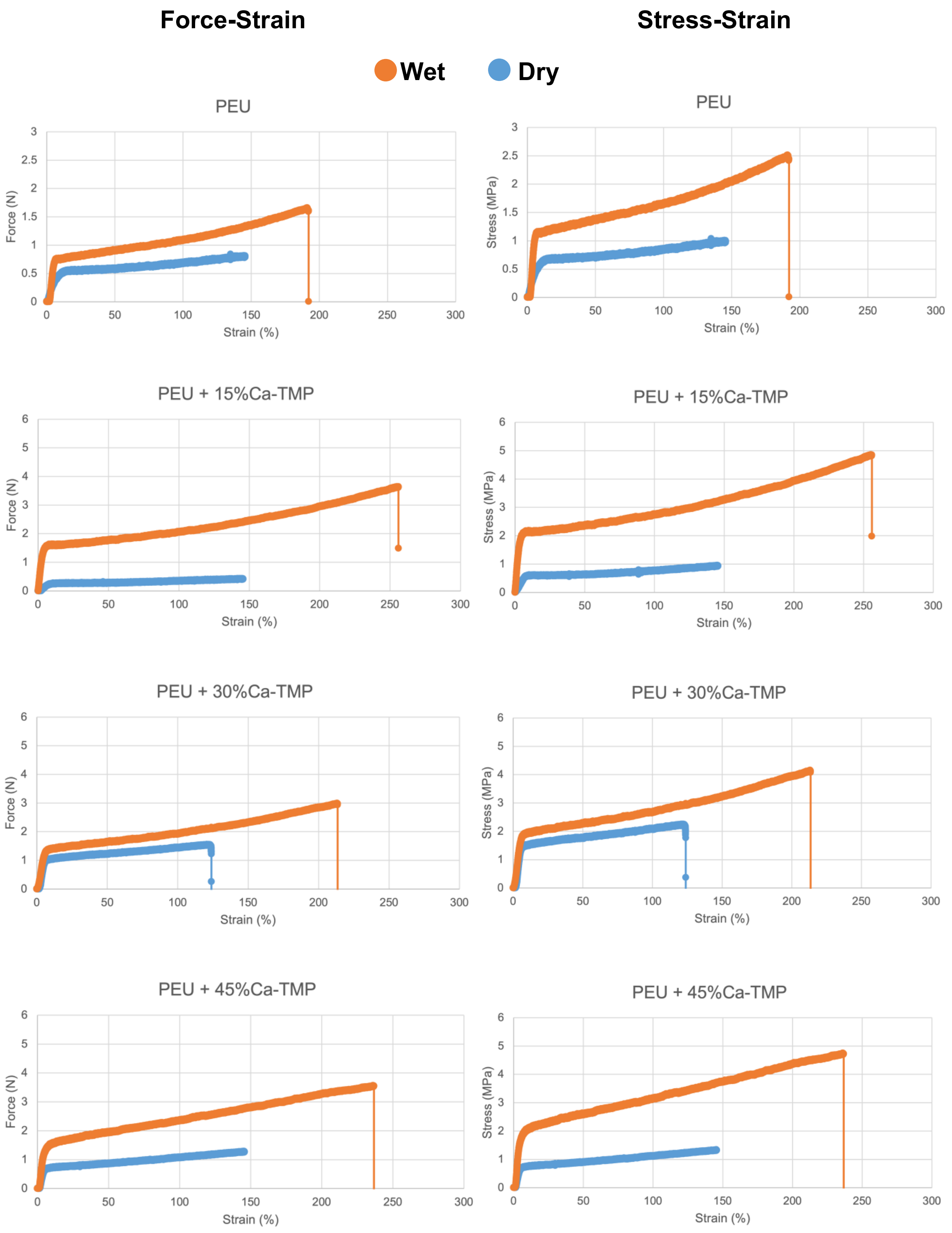

Figure S2: Force-strain and stress-strain curves for all groups in dry and wet conditions.
